# Help-seeking intentions and depression treatment beliefs amongst Sri Lankan Australians: A survey following a mental health literacy framework

**DOI:** 10.1177/13634615241272930

**Published:** 2024-10-30

**Authors:** Amanda Daluwatta, Kathryn Fletcher, Chris Ludlow, Greg Murray

**Affiliations:** 1 Centre for Mental Health, School of Health Sciences, 3783 Swinburne University of Technology, Melbourne, VIC, Australia; 2 Department of Psychological Sciences, School of Health Sciences, 3783 Swinburne University of Technology, Melbourne, VIC, Australia

**Keywords:** Australia, depression, help-seeking intentions, mental health literacy, Sri Lanka, treatment beliefs

## Abstract

There is evidence that Asian migrants in Australia may be relatively reluctant to seek professional help for depression. Reluctance may be related to poor mental health literacy, including limited knowledge of help-seeking options and treatments, and a preference to seek help from informal networks. This study investigated Sri Lankan Australians’ knowledge about managing depression by examining their hypothetical help-seeking intentions and perceptions about interventions and help-providers’ helpfulness. Following Jorm's mental health literacy framework, participants (*N *= 374) were presented with a vignette of a Sri Lankan Australian exhibiting symptomatology consistent with Major Depressive Disorder, and asked to indicate their intentions to seek help by responding to the question: “If you had Mr Silva's problem, what would you do?”. Participants also rated the likely helpfulness of various professional and informal helpers and interventions in addressing a problem akin to Mr Silva’s. Participants reported being likely to seek help from GPs (35.8%), psychologists (25.7%) and friends (24.3%). Additionally, those who intended to seek informal help were significantly less likely to seek professional help, and vice versa. Furthermore, psychologists (94.1%), counsellors (93.3%), close friends (92.5%) and compatriots (91.4%) were most frequently categorised as helpful. Given participants’ high endorsement of psychiatric treatment, psychological treatment, and self-help strategies such as engaging in enjoyable activities, it would be helpful for clinicians to emphasise the benefits of these interventions for managing depression. Additionally, recognising some participants’ inclination towards religious practices and helpers, clinicians can consider integrating these help-seeking behaviours into therapeutic approaches. Future research is warranted to examine the predictors of help-seeking intentions.

## Introduction

While there are now a range of effective interventions and treatments for various mental health conditions (Jorm et al., 2005; Morgan et al., 2019), community members experiencing depression are reluctant to seek professional help (Kohn et al., 2004; Tylee & Jones, 2005; Wang et al., 2007). This reluctance may be more pronounced amongst Asian-born migrants (Lam & Kavanagh, 1996; McDonald & Steel, 1997; Steel et al., 2005), with census data suggesting that Asian-born individuals access subsidised mental health services less frequently than Australian-born residents (4.4% versus 7.8%) (Australian Bureau of Statistics, 2016). Patient file data from Sydney also suggests that South East Asian-born patients consistently reported lower use of a specialised mental health service relative to Australian-born patients (Logan et al., 2017). A multitude of factors can contribute to Asian Australians delaying and underutilising mental health services. One such factor is poor mental health literacy (MHL), which incorporates limited knowledge of help-seeking options, evidence-based practices and self-help strategies (Jorm, 2012), and beliefs about treatments that are at variance with those of health professionals (Jorm et al., 1997b).

Attitudes, knowledge, and beliefs towards different types of professional and informal helpers influence help-seeking practices (ten Have et al., 2010). Jorm (2012) emphasises that the availability of evidence-based, high-quality mental health services alone may not guarantee utilisation by the public. Thus, the decision of the public to seek and utilise such services is contingent upon their belief that these services will help them (Jorm, 2012). For example, a cross-sectional survey found that Australian respondents who believed that a general practitioner (GP) would be unhelpful in the treatment of a mental health concern were 91% less likely to be willing to discuss mental health problems with their GP than those who felt a GP would be helpful (Wrigley et al., 2005). Similarly, perceived helpfulness of interventions impacts help-seeking intentions and treatment compliance (Jorm et al., 1997a). For example, beliefs about the helpfulness of antidepressants was found to predict their use 6 months later in Australian adults (Jorm et al., 2000). A survey in Australian undergraduate students also found a significant relationship between help-seeking intentions and knowledge about the helpfulness of interventions (Smith & Shochet, 2011). Thus, the authors concluded that the awareness of the helpfulness of available interventions seemed likely to increase an individual's willingness to seek help (Smith & Shochet, 2011). Understanding Sri Lankan Australians’ helpfulness beliefs towards different help-providers, interventions, and self-help strategies is crucial in understanding their inclination towards seeking these forms of assistance.

While no study has directly investigated the helpfulness beliefs of depression management options amongst Sri Lankan Australians, numerous empirical studies have examined other Asian Australians’ beliefs about professional and informal help. For example, using Jorm's vignette-based methodology, Wong et al. (2010) found that Chinese-speaking Australian respondents rated professional help, such as doctors (74.0%), counsellors (90.2%), and clinical psychologists (83.6%) as helpful rather than harmful for the person in the depression vignette. Additionally, close family members were provided with a higher helpfulness rating from Chinese-speaking Australians (82.2%) compared to Australian samples (67.9%) (Wong et al., 2010). Although not using the vignette-based methodology, a qualitative study found that Australian residents born in mainland China had (a) limited knowledge of specialist mental health services in the community, (b) limited understanding of the GP’s role in providing mental healthcare and accessing specialist services, and (c) had a strong preference to seek support from Chinese family and friends rather than Western health professionals (Blignault et al., 2008). These findings are consistent with research conducted in Chinese-speaking international students in Australia, who also preferred to seek help from informal sources over professional sources or engage in self-management strategies such as exercise for managing psychological distress (Lu et al., 2014). A cross-sectional survey also reported that young Sri Lankan Australians who were born in Sri Lanka saw significantly less value and need in seeking professional psychological help, relative to young Sri Lankan Australian participants who were born in Australia (Mudunna et al., 2022). Further, Mudunna et al. (2022) found that young Sri Lankan Australian participants who identified as female were more likely to seek mental health care from formal and informal sources, relative to other genders.

When assessing MHL in Asian diaspora communities, it is important to consider that these populations might utilise a range of culturally-sanctioned practices and helpers when managing depression and psychological distress (Amarasuriya et al., 2015a, 2015c). The use and endorsement of traditional practices such as consulting Ayurvedic physicians or engagement in religious rituals, exorcism, and horoscope reading have been observed when managing mental health problems in Sri Lanka (Ediriweera et al., 2012). In Sri Lanka, such practices exist alongside Sri Lankans’ use and endorsement of receiving help from professionals and their informal social network (Ediriweera et al., 2012). In Australia, investigations into Asian migrants’ helpfulness beliefs of culturally specific interventions and professionals have generated mixed findings. A cross-sectional survey in Chinese-speaking Australians found that approximately 30% of respondents rated traditional Chinese medicine doctors, Chinese nutritional foods/supplements and qigong as helpful; whereas only 2.7% rated traditional healers as helpful, and 54.9% suggested the latter could be harmful (Wong et al., 2010). By contrast, a survey of Chinese-speaking international students in Australia found that a high percentage of respondents were uncertain about the helpfulness of some culture-specific types of help such as traditional Chinese medicine doctors for managing psychological distress (Lu et al., 2014); and that respondents endorsed mental health professionals to be more helpful than these culture-specific options (Lu et al., 2014). Beyond these studies, there is limited literature on how perceptions of the wide variety of help-seeking options may influence the help-seeking behaviours of other Asian migrant communities, including Sri Lankan Australians. Although Sri Lankan-born Australians are the 10th largest group of overseas-born residents (Australian Bureau of Statistics, 2022), there is a scarcity of research on their mental health help-seeking beliefs and behaviours. With the Sri Lankan-born population steadily increasing since 2011 (Australian Bureau of Statistics, 2022), there is a distinct need for research seeking to understand Sri Lankan Australians’ beliefs about mental health support including their beliefs about traditional, culture-specific types of help.

### The present study

Following the MHL framework, the present study aimed to investigate Sri Lankan Australians’ knowledge about managing depression by examining their *help-seeking intentions* for managing a hypothetical episode of depression. Knowledge was also assessed by examining participants’ perceptions about the helpfulness of different help-providers and interventions (*helpfulness beliefs*). These two aims answer the following research questions: (1) What course of action would Sri Lankan Australians take if they were personally affected by depression?; (2) Do Sri Lankan Australians prefer professional or informal help-providers when managing depression?; and (3) What interventions and help-providers do Sri Lankan Australians perceive as helpful and unhelpful when managing depression? In line with MHL investigations in Sri Lanka (Amarasuriya et al., 2015b), the assessment of both helpfulness beliefs and help-seeking intentions was considered to provide a more comprehensive understanding of Sri Lankan Australians’ inclination to seek help for depression.

## Method

### Study design

The “Sri Lankan Australians:Let's Talk About Your Mental Health” research project included a cross-sectional survey delivered online via the Qualtrics survey platform. Data collection was between April 2020 and October 2020. Ethical approval was obtained from Swinburne University Human Research Ethics Committee (20202610-4168). All participants received written information about the study and provided consent before proceeding with the survey. Participants were informed that their identity would be anonymous, and that participation was voluntary.

### Participants and recruitment

Individuals were eligible to participate if they were at least 18 years of age, English-speaking, self-identified as having Sri Lankan heritage, currently living in Australia, and had access to the internet. Study recruitment was via a video advertisement containing a link to the survey. The study was advertised via community partners’ mailing lists, websites and social media pages; on Sri Lankan Australian clinicians and leaders’ social media accounts; and through Facebook groups, radio station channels and media organisations that specifically catered to the Sri Lankan Australian population.

### Measures

#### Demographics

Age, gender, education, country of birth, ethnicity, religion, and number of years in Australia were examined.

#### Acculturation

Acculturation refers to a dynamic process that involves changes in an individual's “cultural patterns” including practices, values, and identities arising from sustained intercultural contact, and subsequently affecting the individual's psychological well-being and social functioning (Ward & Geeraert, 2016). Acculturation was measured on a modified version of the Suinn-Lew Asian Self-Identity Acculturation (SL-ASIA) Scale (Suinn et al., 1987). In line with the adaptation for Chinese Australians (Parker et al., 2005), the SL-ASIA was modified for use with Sri Lankan Australians, with the final scale including 12 items. Participants rated each item on a 5-point Likert scale (1 = low; 5 = high) (Suinn et al., 1987). For example, on this sample item, “What language(s) can you speak?”, participants selected one of the following response options; (1) a Sri Lankan language only (e.g.,, Sinhala, Tamil, etc.), (2) mostly a Sri Lankan language, some English, (3) a Sri Lankan language and English about equally well (bilingual), (4) mostly English, some Sri Lankan languages, or (5) only English. In line with the original scale, the modified scale provided a total mean score, with higher scores reflecting higher levels of acculturation to Western or Australian culture (Hsueh et al., 2015), while participants who scored around 3 were considered bicultural, and lower scores reflected Asian or Sri Lankan identification (Hamid et al., 2009).

#### Help-seeking intentions

A culturally adapted vignette was presented of a 30-year-old Sri Lankan Australian named “Mr Silva”, who exhibited symptoms of Major Depressive Disorder consistent with DSM-5 diagnostic criteria. The depression vignette was as follows:

Mr. Silva has been feeling unusually sad and miserable for the last few weeks. Even though Mr. Silva feels tired all the time, Mr. Silva has difficulty falling asleep almost every night. Mr. Silva doesn’t feel like eating and has lost weight. Mr. Silva finds it difficult to concentrate at work and make decisions about day-to-day tasks. This has come to the attention of his boss who is concerned about his lowered productivity. Mr. Silva doesn’t want to socialize with family and friends anymore, and prefers to stay alone all the time. Mr. Silva seems very different to what Mr. Silva was like before. Mr. Silva's wife and friends are very worried about Mr. Silva.

Help-seeking intentions were measured by the open-ended question: “If you had Mr Silva's problem, what would you do?”. To facilitate comparisons with earlier studies (Amarasuriya et al., 2015a; Reavley & Jorm, 2011), responses to this question were coded based on categories that were used in previous surveys, with additional categories formed that were relevant to the Sri Lankan Australian population. In line with the literature (Amarasuriya et al., 2015a), responses were coded with a “yes” or “no” in each category, so that multiple categories were possible. In the current study, more than half (51.1%) of the respondents made more than one help-seeking suggestion. Subsequently, these were regrouped into broader coding categories and responses nominated by more than 1% of the sample are reported below.

#### Helpfulness beliefs of help-providers

**Figure 1.**
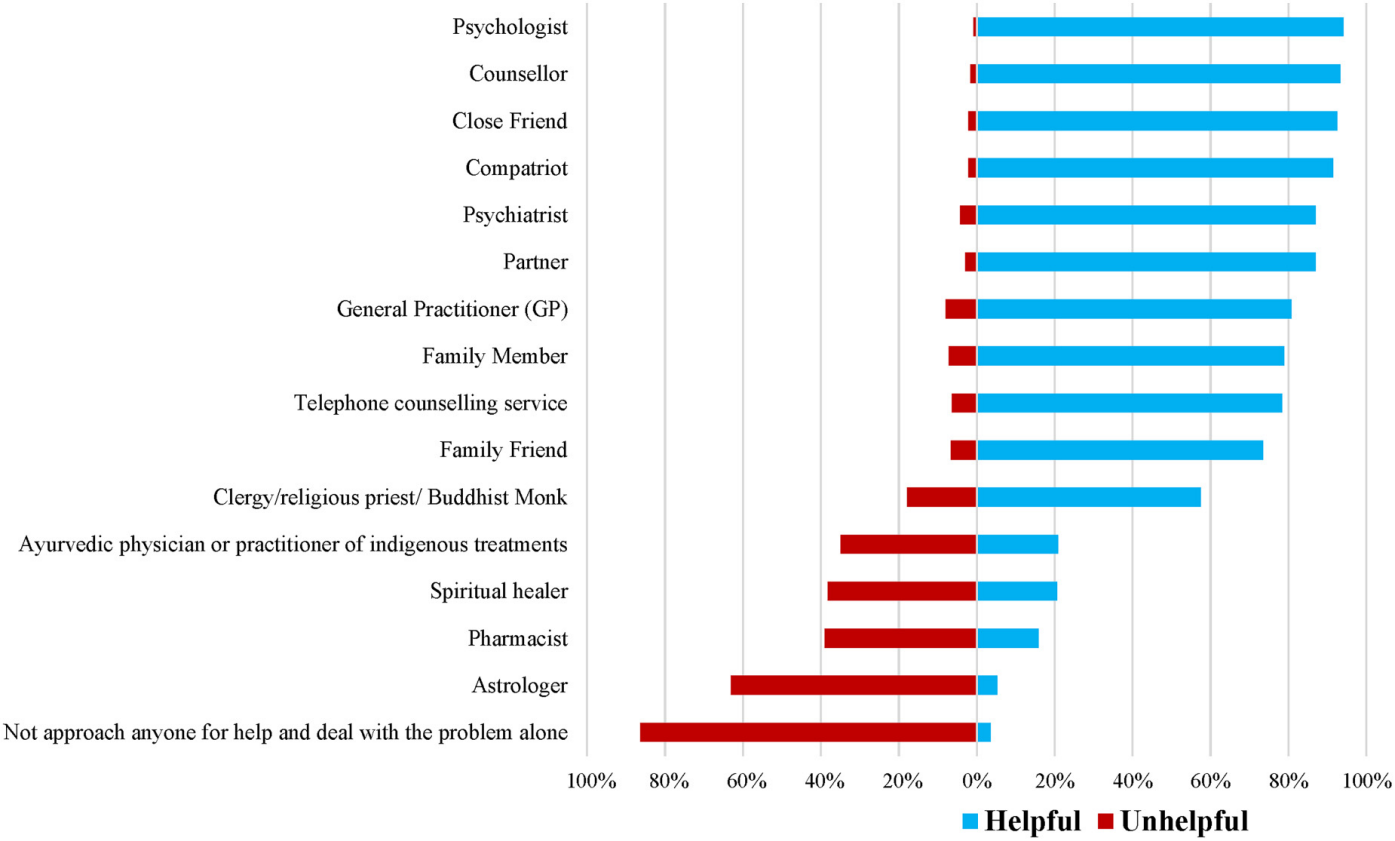
Helpfulness ratings of professional and informal help-providers.

Helpfulness beliefs were examined through participants’ characterisation of the helpfulness of professionals, self-help options and informal sources that could help “Mr Silva” manage the presented problem. In order to make the MHL framework culturally responsive for East Asian migrants (Na et al., 2016), the presented options included diverse forms of help that were relevant to both the Sri Lankan and Australian context. After cultural adaptation of the measure, this section consisted of 16 items (Figure 1). Participants were asked to rate each item as “very helpful”, “fairly helpful”, “neither helpful nor unhelpful”, “fairly unhelpful”, “very unhelpful” or “don’t know”. Responses to the original response categories are presented in the Supplemental Material online. For ease of interpretation, response categories were altered post-hoc as follows: 1. The percentages of the “very helpful” and “fairly helpful” categories were combined to create the category “helpful”; 2. Percentages of the “fairly unhelpful” and “very unhelpful” categories were combined to create the “unhelpful” category; and 3. The “neither helpful nor unhelpful” and “don’t know” options were not presented due to difficulties in interpreting these responses. Frequencies of the new “helpful” and “unhelpful” categories were calculated.

#### Helpfulness beliefs of interventions

**Figure 2.**
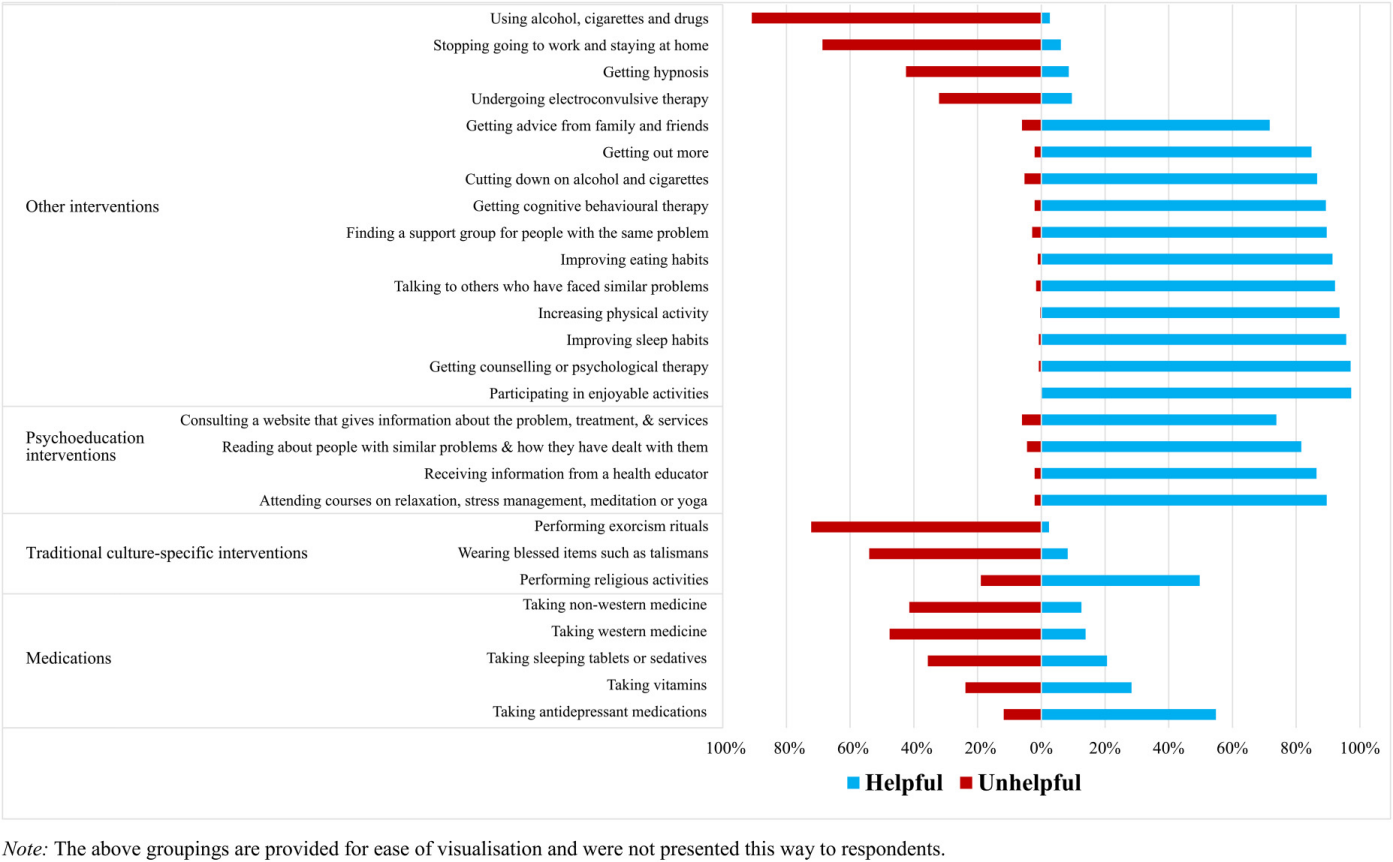
Helpfulness ratings of interventions or actions.

*Note*. The above groupings are provided for ease of visualisation and were not presented this way to respondents.

Participants were provided with a list of actions and interventions that Mr Silva might engage in when trying to deal with his problem and were asked to rate the helpfulness of the presented actions and interventions if they or one of their family members had Mr Silva's problem (Figure 2). For each item, participants could select one of the following response options: “very helpful”, “fairly helpful”, “neither helpful nor unhelpful”, “fairly unhelpful”, “very unhelpful” or “don’t know”. Responses to the original response categories can be found in the Supplemental Material. In line with the above scoring system, frequencies of the new “helpful” and “unhelpful” response categories were calculated.

### Statistical analyses

Statistical analyses were performed using IBM Statistical Package for the Social Sciences (SPSS) Version 27. Due to the descriptive, exploratory nature of this study, valid percent frequencies and 95% confidence intervals were used. Additionally, an aggregate score for all types of informal help that the participants identified as those they would seek if personally affected by the problem was calculated. If the participant intended to seek help from a friend, family member, employee, partner or a person not specified, they were given the response “yes”. If they did not intend to seek help from those informal options, they were given the response “no”. Similarly, an aggregate score for all types of professional help that the participants identified as those they would seek if personally affected by the problem was calculated. The relationship between the aggregates for intentions to seek professional and informal help was tested using the chi-square test of independence.

## Results

### Demographics

In total, *N *= 374 Sri Lankan Australian adults completed the survey. Demographics are presented in Table 1. The mean age was 31.84 years (*SD *= 11.82 years; *range *= 18–77 years). The mean overall acculturation score was 3.02 (*SD *= 0.69; *range *= 1.58–4.58), and most participants reported having an undergraduate degree or higher education level.

**Table 1.** Demographics and other characteristics of the sample (*N* = 374).

Variables	Frequency	%
Gender		
Male	109	29.1
Female	265	70.9
Highest level of education		
Secondary school or equivalent	46	12.3
TAFE course or equivalent	32	8.6
Undergraduate degree	171	45.7
Postgraduate degree	125	33.4
Country of birth		
Australia	117	31.3
Sri Lanka	234	62.6
Other	23	6.1
Years in Australia (*M = *18.02*; SD = *10.08)		
Under 10 years	101	27.0
11 to 20 years	91	24.3
21 to 30 years	155	41.4
Above 31 years	27	7.2
Ethnicity		
Sinhalese	319	85.3
Tamil	24	6.4
Other	31	8.3
Religion		
Buddhist	229	61.2
Christian	24	6.4
Roman Catholic	46	12.3
Atheist or Agnostic	43	11.5
Other	32	8.6

### Research Question 1: Help-seeking intentions

Table 2 displays Sri Lankan Australians’ intended help-seeking actions if they had Mr Silva's problem. The final coding categories are presented in Table 2. The most frequently mentioned help-seeking intention was “help from a GP or doctor” (35.8%), followed by “help from a psychologist” (25.7%) and “help from a friend” (24.3%). Seeking help online, managing work, and getting out more were the least rated, with between 1.3% and 1.9% of respondents nominating them as likely help-seeking actions.

**Table 2.** Help-seeking intentions if personally affected by the problem in the vignette (*N *= 374).

Intended help-seeking actions	Percentage intending to seek help (95% Confidence Intervals)
Professional options	
Help from a General Practitioner (GP)/doctor	35.8 [30.7, 40.9]
Help from a psychologist	25.7 [21.7, 30.2]
Help from a psychiatrist/specialist	9.6 [7.0, 12.8]
Help from a counsellor	9.6 [7.0, 12.6]
Informal options	
Help from a friend	24.3 [20.3, 28.6]
Help from a person not specified	20.1 [16.0, 24.3]
Help from a family member	15.8 [12.0, 19.3]
Help from one's partner	6.4 [3.8, 9.1]
Help from an employer and/or work colleague	2.1 [0.8, 3.7]
Self-management strategies	
Active coping	10.4 [7.2, 13.6]
Understanding the problem	6.1 [4.0, 8.8]
Avoidant coping	5.6 [3.5, 8.0]
Dealing with it alone	3.5 [1.9, 5.3]
Religious-oriented strategies	3.2 [1.6, 5.1]
Other	2.1 [0.8, 3.7]
Managing work	1.9 [0.5, 3.2]
Seeking help online	1.6 [0.5, 2.9]
Don’t know	1.3 [0.3, 2.7]
Getting out more or being more social	1.3 [0.3, 2.7]

*Note:* The “avoidant coping” category captured a range of heterogeneous behaviours related to substance use, suicidal ideation, ignoring or minimising the problem, and isolating oneself. The “active coping” category included behaviours such as engaging in exercise, relaxation activities and taking a holiday. All responses not relevant to the 18 categories were included in the “other” category.

### Research Question 2: Professional vs informal help-seeking intentions

Overall, 53.7% of the sample indicated that they would intend to use some type of professional help option, while 50.3% indicated intentions to use some type of informal help. Results indicated that those who intended to seek informal help were significantly less likely to also seek professional help and vice versa (Pearson chi-square χ(1) = 29.17, *p* < .001).

### Research Question 3a: Beliefs about the likely helpfulness of help-providers

Shown in Figure 1 are the helpfulness versus unhelpfulness ratings for each of the different help-providers (95% confidence intervals are provided in the Supplemental Material online). The help-providers most commonly described as helpful were psychologists (94.1%), closely followed by counsellors (93.3%), close friends (92.5%) and compatriots (a person with lived experience of the problem, 91.4%). Astrologers (63.1%) and pharmacists (39.0%) were rated as the most unhelpful sources of help. Respondents also rated dealing with the problem alone and not approaching anyone for help (86.4%) as unhelpful.

### Research Question 3b: Beliefs about the likely helpfulness of interventions and actions

Figure 2 presents the helpfulness versus unhelpfulness ratings for each action or intervention (95% confidence intervals are provided in the Supplemental Material). Participating in enjoyable activities (97.3%), attending counselling or psychological therapy (97.1%), improving sleeping habits (95.7%), increasing physical activity (93.6%) and talking to others who have faced similar problems (92.2%) were the interventions and actions most frequently categorised as helpful. In comparison, using substances (90.9%) and performing exorcism rituals (72.2%) were considered the most unhelpful.

## Discussion

To our knowledge, this is the first study to comprehensively examine Sri Lankan Australians’ personal help-seeking intentions and helpfulness beliefs about different types of interventions and help-providers for depression management. A vignette-based assessment of intention to seek help suggested that Sri Lankan Australians preferred GPs and psychologists among healthcare professionals. When asked about people and interventions that would be helpful, participants also showed a high endorsement of psychologists, counsellors, close friends and psychologically-oriented interventions. Following is a more in-depth discussion of the findings.

### Research Question 1: Help-seeking intentions for managing a hypothetical episode of depression

The present study suggests that Sri Lankan Australians would be likely to seek help from a GP if personally affected by depression. Our findings broadly align with the National Survey of Mental Health Literacy and Stigma, whereby Australian respondents also believed that GPs would be the most helpful (Reavley & Jorm, 2011). Both Sri Lankan Australian and Australian respondents’ attitudes align with the healthcare system in Australia, where GPs are the first point of contact for mental health support in the community and are considered the gateway to other services (Jorm et al., 2005; Wong et al., 2010).

Consistent with MHL studies conducted in Sri Lanka (Amarasuriya et al., 2015a, 2015b), this sample of Sri Lankan Australians also reported that they would seek help from a friend if they experienced depression. Participants also strongly endorsed informal sources, such as close friends, compatriots and partners, as helpful sources of support. Our findings highlight the important role that Sri Lankan Australians’ informal social network may play, suggesting that informal social networks should be provided with mental health first-aid training (Amarasuriya et al., 2015b, 2018).

### Research Question 2: Preference for professional or informal help-providers

A slightly higher proportion of participants indicated that they would seek help from professional help-providers (53.7%) rather than from informal help-providers (50.3%). These findings are inconsistent with studies conducted in Sri Lanka, which found participants were more likely to seek help from informal sources such as friends and parents than from professional sources when personally dealing with the problem in the vignette (Amarasuriya et al., 2015a).

Interestingly, our findings also suggested that participants who intended to seek informal help were significantly less likely to also seek professional help, and vice versa. These findings suggest a reluctance for some to seek professional help and point to the need to examine the underlying reasons for this. Mistrust of mental health providers and concerns about confidentiality have been consistently reported as significant barriers to effective engagement with mental health services amongst culturally and linguistically diverse community members (Radhamony et al., 2023), Asian Australians (Wynaden et al., 2005) and Chinese Australians (Blignault et al., 2008; Choi et al., 2015). In light of this, a recent systematic review focusing on British South Asian mental health service users suggested the importance of a sensitive approach that emphasised a commitment to confidentiality (Prajapati & Liebling, 2022). The review also recommended that service providers prioritise building trust and ensuring cultural safety for South Asian service users in the United Kingdom (Prajapati & Liebling, 2022). Future studies could examine whether such reluctance by some Sri Lankan Australians is associated with perceived negative consequences of seeking help such as a lack of confidentiality.

### Research Question 3: Helpfulness beliefs of help-providers and interventions

Although participants reported an intention to seek help from GPs, they perceived GPs (80.7%) as less helpful than psychologists (94.1%) and counsellors (93.3%) when evaluating the helpfulness of different help-providers for a problem akin to Mr Silva's. These results diverge from data collected in the National Survey of Mental Health Literacy and Stigma, which found that GPs (90.0%) received the highest helpfulness rating among the people who could help for the person in the depression vignette (Reavley & Jorm, 2011). However, our participants’ high helpfulness rating of counsellors is comparable to findings in Chinese-speaking Australian samples, who also preferred counselling professionals (e.g., counsellors, social workers) to other professionals for the person in the depression vignette (Wong et al., 2010). These findings may reflect common aetiological beliefs held amongst the Chinese and Sri Lankan populations in Australia. Specifically, research has suggested that Asian migrant communities tend to believe that depression is caused by situational and contextual factors such as life stressors and interpersonal problems (Antoniades et al., 2017; Wong et al., 2010). Thus, individuals with psychosocial problems may be seen as needing assistance from professionals in supportive roles, who can listen and give advice (Jorm et al., 2005). Given these findings about the perceived helpfulness of GPs compared to counselling professionals, it is essential to educate GPs about this migrant group's preferred choice of professional so that prompt referrals can be made.

Participants rated a range of evidence-based self-help interventions as likely to be helpful, such as participating in enjoyable activities, increasing physical activity, improving sleep and talking to others who have encountered similar problems. Whereas unhelpful ratings were most common for stopping going to work and staying at home, performing exorcism rituals, using substances, and wearing blessed items such as talismans. These beliefs about interventions broadly align with evidence-based practice for the management of depression (Morgan & Jorm, 2008), supporting the potential use of these strategies in behavioural activation (Amarasuriya et al., 2015a) with Sri Lankan Australians. Additionally, participants endorsed more positive beliefs towards lifestyle interventions than towards medical interventions, consistent with previous surveys of general Australian (Jorm et al., 2005; Reavley & Jorm, 2011), Chinese-speaking Australian (Wong et al., 2010) and Sri Lankan populations (Amarasuriya et al., 2015a).

Among medications, the present Sri Lankan Australian sample considered antidepressants as the most helpful for depressive symptoms. Respondents’ helpfulness rating of antidepressants (54.8%) was higher than helpfulness ratings found in Chinese-speaking Australians (40.9%), Japanese populations (34.8%) (Wong et al., 2010) and Sri Lankan populations (29.0%) (Amarasuriya et al., 2015a). Further, a qualitative study in Sri Lankan Australians with lived experience of depressive symptoms found that respondents were reluctant to engage with pharmacological interventions (Antoniades et al., 2017). It is possible that the current participants’ high endorsement of antidepressants may be related to our sample having lived in Australia for an average of more than 18 years, and thus being exposed to the many community interventions that have been implemented in Australia over the past 25 years to improve MHL (Jorm, 2021), resulting in more positive attitudes towards medication. Nonetheless, these results are promising in terms of suggesting Sri Lankan Australians’ preparedness to engage with medical interventions.

A strength of the current study was the incorporation of culture-specific help-providers and interventions that are commonly found in Sri Lanka. In line with findings from Sri Lankan (Amarasuriya et al., 2015a) and Chinese-speaking Australian samples (Wong et al., 2010), participants perceived religious figures and performing religious activities as helpful rather than harmful. The high endorsement of the clergy and religious strategies suggests that Asian diaspora communities may perceive such help as being integral to the healing process (Amarasuriya et al., 2015a). However, in Western countries, mental health professionals tend to be less religious than their clients; with a qualitative study in the Netherlands finding that many professionals in a secular clinic tended to ignore and remain unaware of consumers’ religion or spirituality, and sometimes would also deliberately avoid the topic (Van Nieuw Amerongen-Meeuse et al., 2018). Preliminary evidence suggests that faith-based cognitive behaviour therapy might be helpful for those with depression who have religious or spiritual beliefs (Morgan et al., 2019). It may therefore be beneficial for health professionals to be more aware of how their patient's beliefs can diverge greatly from their own (Jorm et al., 1997b), and perhaps even involve these religious help-providers as partners in community-based care initiatives.

### Discrepancies between help-seeking intentions and helpfulness beliefs

Participants provided high helpfulness ratings for psychologists, counsellors and participating in counselling and psychological therapies. Despite this, their reported intentions to seek help from psychologists (25.7%) and counsellors (9.6%) was considerably lower. Discrepancies between perceived helpfulness and intended use are consistent with other research, whereby interventions involving mental health professionals were often rated as helpful, but were rarely used in practice (Jorm et al., 2000).

There are several possible reasons for these discrepancies. First, reluctance to seek assistance may be related to more prominent attitudes and beliefs, such as stigma or the belief that mental health professional services are a scarce resource that is generally only available to people with severe conditions (Jorm et al., 2000). Second, subtle differences in data collection between the questions may contribute to this discrepancy. We used an open-ended format to collect responses to the help-seeking intentions question, and asked participants to answer the question in relation to themselves. By contrast, a fixed rating scale was used to examine the perceived helpfulness of help-providers and interventions, and participants were asked to respond in relation to themselves or one of their family members. It is possible that findings for the help-seeking intentions question might have been higher if fixed rating scale items had been presented instead of an open-ended question (Amarasuriya et al., 2015b). However, this methodology has been suggested as more accurately reflecting cognitive mechanisms if affected by depression and deciding on the type of help to seek (Amarasuriya et al., 2015b). Hechanova and Waelde (2017) suggest that within Asian collectivistic cultures, healing is a consequence of interdependence, highlighting that the mental health of the group is at least as significant to the individual as their own health. Additionally, within cultures exhibiting a strong collectivist culture, community members are encouraged to actively support individuals within the in-group, including those individuals experiencing mental health conditions (Natalia & Fridari, 2022). Therefore, it is possible that we have tapped into social, collective norms related to Sri Lankan Australians perceiving help-seeking options as more beneficial for their family rather than for themselves. Further extension of this work would benefit from refining the current methodology to understand these differences.

### Clinical implications

The study's findings have some important clinical implications. Encouragingly, most standard psychological and psychiatric treatments were rated as helpful rather than unhelpful, including behavioural interventions, and taking antidepressant medications. By contrast, unhelpful coping strategies, such as stopping going to work and using substances were rated as unhelpful, which aligns with expert opinion and clinical guidelines (Amarasuriya et al., 2015a; Morgan et al., 2013). Additionally, Sri Lankan Australian participants also endorsed religious figures as helpful more frequently than unhelpful, however, psychologists and counsellors were still identified as the most helpful agents. Taken together, these results suggest the existence of a complex, pluralistic mental health belief system, including the co-existence of biomedical and religious beliefs (May et al., 2014) among Sri Lankan Australians; which in turn could create an opportunity for Western mental health services and religious practitioners to service this community within a holistic and culturally responsive model (May et al., 2014). For example, this might involve transcultural mental health services acknowledging the existence of both conventional and traditional mental health interventions (May et al., 2014), such as Sri Lankan Australians’ connection to their spirituality, and how this connection supports their wellbeing and recovery.

### Limitations

Study findings need to be considered in light of several methodological limitations. First, the survey was titled “Sri Lankan Australians: Let's Talk About Your Mental Health”, possibly priming participants to the specific focus of the study and subsequently influencing their responses in the open-ended questions. Second, the current sampling method poses a threat of selection bias (Lu et al., 2014), and it is possible that community members with a greater knowledge of, and interest in, mental health issues were more inclined to participate. Therefore, future research could use more robust sampling strategies that would strengthen the generalisability of findings to the broader Sri Lankan Australian population. Third, it is crucial to bear in mind when interpreting these findings that the sample comprised of highly educated Sri Lankan Australians, with 87.7% of participants having attained tertiary education or equivalent. Research has suggested that educational attainment is a significant predictor of attitudes towards help-seeking for mental health concerns (Tuazon & Clemente, 2022). Additionally, the mean overall acculturation score suggests that, on average, participants in the current study perceived themselves as bicultural. Hamid et al. (2009) found a significant positive relationship between Asian Australian university students’ level of behavioural acculturation and their attitudes towards seeking professional psychological assistance. Therefore, the current results may not generalise to other samples of Sri Lankan Australians who have different acculturation levels and educational backgrounds. To identify those who are less likely to seek professional help, future studies could examine the associations between personal characteristics such as acculturation levels of Sri Lankan Australians, and their intentions to seek help for depression.

Finally, despite mental health literacy's established utility as a structure for understanding factors that influence individuals’ mental health help-seeking (Renwick et al., 2024; Spiker & Hammer, 2019), Jorm's mental health literacy concept and vignette-based methodology has been criticised (Kutcher et al., 2016) for lacking ecological validity (Furnham & Swami, 2018) and for promoting the psychiatric and biogenetic conceptualisation of mental illness (Mansfield et al., 2020). Another important methodological limitation of the present study was that respondents were asked to identify the condition in the vignette, which then formed the basis for their knowledge or attitudes about available help-providers or treatments. A respondent could incorrectly label the person in the vignette as having a “physical health disorder”, and subsequently provide personal help-seeking intentions and treatment beliefs related to this label. Arguably, this different label will likely result in substantially different responses, for example, the respondent could provide low helpfulness ratings for the use of an antidepressant medication (O’Connor et al., 2014). Thus, some participants may have provided answers to the personal help-seeking intentions and treatment belief questions that may not actually reflect their knowledge of the treatment of depression (O’Connor et al., 2014). As such, it may be useful for future research to not use the vignette-based method, but instead utilise a scale-based measure of mental health literacy (O’Connor & Casey, 2015).

## Conclusions

This study was the first to comprehensively examine help-seeking intentions and helpfulness beliefs for the general Sri Lankan community in Australia. Within its limitations, study findings suggest that Sri Lankan Australians are willing to approach professional help providers, and that close friends remain an important source of support. Results also suggested that most participants reported helpfulness beliefs that were congruent with evidence-based practices. Additionally, some respondents rated traditional sources such as religious figures as likely to be helpful. Campaigns to increase the MHL of Sri Lankan Australians need to consider these beliefs so that culturally relevant psychoeducation programs can be developed (Wong et al., 2010). Future research is warranted to examine the predictors of such help-seeking intentions in order to identify those who are less likely to seek professional help.

## Supplemental Material

sj-pdf-1-tps-10.1177_13634615241272930 - Supplemental material for Help-seeking intentions and depression treatment beliefs amongst Sri Lankan Australians: A survey following a mental health literacy framework Supplemental material, sj-pdf-1-tps-10.1177_13634615241272930 for Help-seeking intentions and depression treatment beliefs amongst Sri Lankan Australians: A survey following a mental health literacy framework by Amanda Daluwatta, Kathryn Fletcher, Chris Ludlow, and Greg Murray in Transcultural Psychiatry
