# Supplementary material for: Help-seeking intentions and depression treatment beliefs amongst Sri Lankan Australians: A survey following a mental health literacy framework
Source: Transcult Psychiatry. 2024 Oct 30;62(3):354–65. doi: 10.1177/13634615241272930 (PMC12171073; doi:10.1177/13634615241272930)
Supplement: sj-pdf-1-tps-10.1177_13634615241272930 - Supplemental material for Help-seeking intentions and depression treatment beliefs amongst Sri Lankan Australians: A survey following a mental health literacy framework [file sj-pdf-1-tps-10.1177_13634615241272930.pdf]

Help-seeking intentions and depression treatment beliefs amongst Sri Lankan Australians: A survey following a mental health literacy framework

Amanda Daluwatta, Kathryn Fletcher, Chris Ludlow & Greg Murray

Supplemental material

Table S1. Helpfulness rating of professional & informal sources of help using original response options (*N* = 374)

Help-provider	Rated as "Very Helpful"		Rated as "Fairly Helpful"		Rated as "Neither helpful nor unhelpful"		Rated as "Fairly Unhelpful"		Rated as "Very Unhelpful"		Rated as "Don't Know"	
	%	(95% CIs)	%	(95% CIs)	%	(95% CIs)	%	(95% CIs)	%	(95% CIs)	%	(95% CIs)
General Practitioner or Family Doctor	38.8	(33.8-43.5)	42.0	(36.8-46.9)	8.6	(5.7-11.6)	6.1	(3.8-8.8)	1.9	(0.5-3.4)	2.7	(1.3-4.3)
Pharmacist	1.9	(0.5-3.3)	13.9	(10.5-17.9)	35.0	(30.1-39.8)	20.6	(16.7-24.7)	18.4	(14.7-22.5)	10.2	(7.2-13.4)
Psychiatrist	56.4	(51.2-61.4)	30.5	(26.0-35.1)	4.3	(2.4-6.5)	3.7	(2.1-5.8)	0.5	(0.0-1.4)	4.5	(2.7-6.7)
Psychologist	72.5	(68.1-77.0)	21.7	(17.7-25.7)	2.4	(1.1-4.0)	0.8	(0.0-1.9)	-	-	2.7	(1.3-4.3)
Counsellor	68.4	(63.4-73.4)	24.9	(20.8-29.5)	2.4	(1.1-4.0)	0.5	(0.0-1.4)	1.1	(0.3-2.2)	2.7	(1.1-4.5)
Telephone counselling service	41.2	(36.2-46.6)	37.2	(32.4-42.1)	9.6	(6.7-12.6)	3.5	(1.9-5.6)	2.9	(1.3-4.7)	5.6	(3.4-8.2)
Clergy/religious priest/ Buddhist Monk	19.8	(15.9-24.0)	37.7	(32.5-42.7)	19.5	(15.8-23.3)	8.0	(5.5-10.8)	9.9	(6.8-12.9)	5.1	(2.9-7.4)
Ayurvedic physician or practitioner of indigenous treatments	3.2	(1.6-5.2)	17.6	(13.6-21.5)	29.7	(25.4-34.3)	13.9	(10.6-17.6)	21.1	(16.9-25.1)	14.4	(10.9-18.0)
Spiritual healer	2.9	(1.3-4.6)	17.6	(13.7-21.7)	28.6	(23.8-33.0)	13.1	(9.7-16.8)	25.1	(21.1-29.6)	12.6	(9.3-16.1)
Astrologer	0.5	(0.0-1.3)	4.8	(2.9-7.0)	19.3	(15.3-23.5)	13.6	(10.7-17.2)	49.5	(44.3-54.8)	12.3	(9.1-15.7)
Family Member	34.2	(29.4-38.9)	44.7	(39.4-49.6)	12.6	(9.1-16.2)	3.7	(2.1-6.1)	3.5	(1.9-5.4)	1.3	(0.3-2.6)
Partner	48.9	(43.7-54.1)	38.0	(33.2-42.8)	6.7	(4.3-9.4)	1.3	(0.3-2.7)	1.6	(0.5-3.0)	3.5	(1.9-5.3)
Family Friend	27.5	(22.9-32.1)	46.0	(41.0-51.2)	16.6	(13.1-20.4)	3.5	(1.6-5.4)	3.2	(1.6-5.0)	3.2	(1.6-5.2)
Close Friend	48.7	(43.7-53.9)	43.9	(38.6-49.1)	4.5	(2.7-6.8)	0.8	(0.0-1.9)	1.3	(0.3-2.5)	0.8	(0.0-1.9)
Compatriot	46.3	(40.8-51.6)	45.2	(40.2-50.7)	3.7	(2.1-5.9)	1.6	(0.5-3.0)	0.5	(0.0-1.4)	2.7	(1.1-4.3)
Not approach anyone for help and deal with the problem alone	1.3	(0.3-2.5)	2.1	(0.8-3.8)	7.2	(4.6-9.6)	7.5	(4.8-10.4)	78.9	(74.8-82.9)	2.9	(1.3-4.6)

Help-seeking intentions and depression treatment beliefs amongst Sri Lankan Australians: A survey following a mental health literacy framework

Amanda Daluwatta, Kathryn Fletcher, Chris Ludlow & Greg Murray

Table S2. Helpfulness rating of each type of intervention (N = 374)

Interventions	Rated as "Very Helpful" % (95% CIs)	Rated as "Fairly Helpful" % (95% CIs)	Rated as "Neither helpful nor unhelpful" % (95% CIs)	Rated as "Fairly Unhelpful" % (95% CIs)	Rated as "Very Unhelpful" % (95% CIs)	Rated as "Don't Know" % (95% CIs)
Medications						
Taking western medicine	2.4 (1.1-4.0)	11.5 (8.2-14.9)	29.4 (24.5-34.3)	14.7 (11.2-18.5)	32.9 (28.0-37.6)	9.1 (6.4-12.1)
Taking non-western medicine	1.6 (0.5-2.9)	11.0 (7.7-14.5)	33.4 (28.6-38.8)	15.0 (11.5-18.7)	26.5 (22.0-30.9)	12.6 (9.3-16.0)
Taking antidepressant medication	18.2 (14.4-22.2)	36.6 (31.3-41.4)	16.8 (13.4-20.9)	6.1 (3.8-8.6)	5.6 (3.2-8.0)	16.6 (13.1-20.9)
Taking sleeping tablets or sedatives	3.2 (1.6-5.1)	17.4 (13.6-21.4)	26.5 (22.2-31.0)	16.8 (13.1-20.6)	18.7 (15.0-22.7)	17.4 (13.4-21.1)
Taking vitamins	5.1 (2.9-7.3)	23.3 (18.9-27.7)	39.0 (34.0-44.1)	11.2 (8.0-14.5)	12.6 (9.3-16.1)	8.8 (6.2-12.0)
Traditional interventions						
Performing religious activities	14.2 (10.9-17.9)	35.6 (31.2-40.5)	25.9 (21.4-30.2)	9.4 (6.4-12.4)	9.6 (6.6-12.5)	5.3 (3.2-7.8)
Performing exorcism rituals	0.3 (0.0-0.8)	2.1 (0.8-3.7)	13.1 (9.9-16.9)	8.6 (5.9-11.5)	63.6 (58.7-68.4)	12.3 (9.1-15.7)
Wearing blessed items such as talismans	1.3 (0.3-2.6)	7.0 (4.5-9.9)	25.9 (21.4-30.4)	9.6 (6.6-12.9)	44.4 (38.9-49.5)	11.8 (8.5-15.0)
Psychoeducation interventions						
Reading about people with similar problems and how they have dealt with them	21.1 (17.1-25.4)	60.4 (55.3-65.8)	11.8 (8.6-15.0)	3.2 (1.6-5.1)	1.3 (0.3-2.4)	2.1 (0.8-3.7)
Attending courses on relaxation, stress management, meditation or yoga	41.2 (36.4-46.0)	48.4 (43.6-53.7)	7.2 (4.8-10.2)	1.1 (0.3-2.1)	1.1 (0.0-2.1)	1.1 (0.3-2.1)
Consulting a website that gives information about the problem, its treatment, and available services	28.3 (24.1-33.2)	45.5 (40.4-50.5)	15.8 (12.3-19.5)	4.0 (2.1-6.1)	2.1 (0.8-3.7)	4.3 (2.4-6.4)

Receiving information from a health educator	41.7 (36.9-46.8)	44.7 (39.6-49.5)	8.6 (5.9-11.5)	0.8 (0.0-1.9)	1.3 (0.3-2.7)	2.9 (1.3-4.8)
Other interventions						
Increasing physical activity	55.9 (50.8-61.0)	37.7 (32.4-42.6)	5.9 (3.7-8.5)	-	0.3 (0.0-0.8)	0.3 (0.0-0.8)
Improving sleep habits	64.4 (59.6-69.4)	31.3 (26.3-35.9)	2.9 (1.4-4.8)	0.5 (0.0-1.3)	0.3 (0.0-0.8)	0.5 (0.0-1.4)
Participating in enjoyable activities	69.5 (64.3-74.6)	27.8 (23.0-32.6)	2.4 (0.8-4.0)	-	-	0.3 (0.0-0.8)
Getting out more	38.8 (34.0-43.7)	46.0 (40.8-51.1)	11.0 (7.9-14.2)	1.9 (0.8-3.3)	0.3 (0.0-0.8)	2.1 (0.8-3.7)
Getting advice from family and friends	21.4 (17.4-25.4)	50.3 (45.1-55.6)	20.6 (16.5-24.8)	4.0 (2.1-5.9)	2.1 (0.8-3.8)	1.6 (0.3-2.9)
Getting hypnosis	1.6 (0.5-2.9)	7.0 (4.5-9.6)	20.3 (16.0-24.9)	11.8 (8.6-15.2)	30.7 (26.2-35.3)	28.6 (23.8-33.2)
Getting counselling or psychological therapy	72.5 (67.8-76.9)	24.6 (20.0-29.1)	1.1 (0.3-2.4)	0.5 (0.0-1.3)	0.3 (0.0-0.8)	1.1 (0.3-2.2)
Getting cognitive behavioural therapy	52.4 (47.3-57.2)	36.9 (32.4-42.0)	4.5 (2.4-6.7)	1.9 (0.5-3.2)	0.3 (0.0-0.8)	4.0 (2.1-6.1)
Talking to others who have faced similar problems	43.9 (39.0-49.2)	48.4 (43.3-53.5)	5.6 (3.5-8.0)	1.1 (0.3-2.1)	0.5 (0.0-1.3)	0.5 (0.0-1.3)
Improving eating habits	46.5 (41.7-51.6)	44.9 (39.8-49.7)	6.1 (3.7-8.8)	0.8 (0.0-1.9)	0.3 (0.0-0.8)	1.3 (0.3-2.4)
Undergoing electroconvulsive therapy	1.9 (0.5-3.2)	7.8 (4.8-10.4)	19.8 (15.8-23.8)	7.8 (5.3-10.7)	24.3 (20.3-28.9)	38.5 (33.7-43.6)
Cutting down on alcohol and cigarettes	50.0 (45.2-55.3)	36.6 (31.6-41.2)	6.4 (4.0-9.1)	2.4 (1.1-4.0)	2.9 (1.3-4.8)	1.6 (0.5-2.9)
Finding a support group for people with the same problem	43.3 (38.5-48.7)	46.3 (41.4-51.1)	5.1 (3.2-7.5)	1.9 (0.8-3.5)	1.1 (0.3-2.1)	2.4 (1.1-4.0)
Stopping going to work and staying at home	1.3 (0.3-2.7)	4.8 (2.9-7.1)	20.3 (16.4-24.3)	29.9 (25.3-34.6)	38.8 (33.9-43.3)	4.8 (2.7-7.1)
Using alcohol, cigarettes and drugs	0.8 (0.0-1.9)	1.9 (0.8-3.5)	4.3 (2.4-6.5)	11.2 (8.3-14.7)	79.7 (75.5-83.7)	2.1 (0.8-3.7)

Help-seeking intentions and depression treatment beliefs amongst Sri Lankan Australians: A survey following a mental health literacy framework

Amanda Daluwatta, Kathryn Fletcher, Chris Ludlow & Greg Murray

Table S3. Helpful and unhelpful ratings of professional and informal sources of help (*N* = 374)

Help-provider	Rated as “Helpful”		Rated as "Unhelpful"	
	%	(95% CIs)	%	(95% CIs)
General Practitioner (GP) or Family Doctor	80.7	(76.7-84.8)	8.0	(5.3-10.7)
Pharmacist	15.8	(12.0-19.8)	39.0	(33.7-44.1)
Psychiatrist	86.9	(83.7-90.1)	4.3	(2.4-6.4)
Psychologist	94.1	(91.7-96.5)	0.8	(0.0-1.9)
Counsellor	93.3	(90.6-95.7)	1.6	(0.5-2.9)
Telephone counselling service	78.3	(74.1-82.4)	6.4	(4.0-8.8)
Clergy/religious priest/Buddhist Monk	57.5	(52.7-62.6)	17.9	(14.2-21.9)
Ayurvedic physician or practitioner of indigenous treatments	20.9	(16.6-25.7)	35.0	(30.2-40.1)
Spiritual healer	20.6	(16.3-24.9)	38.2	(33.2-43.3)
Astrologer	5.3	(3.2-7.8)	63.1	(58.3-67.9)
Family Member	78.9	(74.9-82.9)	7.2	(4.6-9.9)
Partner	86.9	(83.4-90.4)	2.9	(1.3-4.8)
Family Friend	73.5	(68.5-77.8)	6.7	(4.3-9.4)
Close Friend	92.5	(89.6-95.2)	2.1	(0.8-3.7)
Compatriot	91.4	(88.5-94.1)	2.1	(0.8-3.5)
Not approach anyone for help and deal with the problem alone	3.5	(1.9-5.3)	86.4	(83.2-89.6)

Note: Percentages do not total to 100% due to not including the “don’t know” and “neither helpful nor unhelpful” responses in the table

Help-seeking intentions and depression treatment beliefs amongst Sri Lankan Australians: A survey following a mental health literacy framework

Amanda Daluwatta, Kathryn Fletcher, Chris Ludlow & Greg Murray

Table S4. Helpful and unhelpful ratings of each type of intervention (N = 374)

Interventions	Rated as "Helpful"		Rated as "Unhelpful"	
	%	(95% CIs)	%	(95% CIs)
Medications				
Taking western medicine	13.9	(10.4-17.4)	47.6	(42.5-52.1)
Taking non-western medicine	12.6	(9.4-15.8)	41.4	(36.4-46.3)
Taking antidepressant medications	54.8	(49.5-59.6)	11.8	(8.6-15.0)
Taking sleeping tablets or sedatives	20.6	(16.6-24.6)	35.6	(31.0-40.4)
Taking vitamins	28.3	(23.8-33.4)	23.8	(19.8-28.3)
Traditional culture-specific interventions				
Performing religious activities	49.7	(44.4-54.8)	19.0	(15.0-23.0)
Performing exorcism rituals	2.4	(1.1-4.3)	72.2	(67.6-76.7)
Wearing blessed items such as talismans	8.3	(5.9-11.0)	54.0	(48.4-59.6)
Psychoeducation interventions				
Reading about people with similar problems and how they have dealt with them	81.6	(77.3-85.8)	4.5	(2.4-6.7)
Attending courses on relaxation, stress management, meditation or yoga	89.6	(86.4-92.5)	2.1	(0.8-3.7)
Consulting a website that gives information about the problem, its treatment, and available services	73.8	(69.3-78.1)	6.1	(4.0-8.6)
Receiving information from a health educator	86.4	(82.9-89.6)	2.1	(0.8-3.7)
Other interventions				
Increasing physical activity	93.6	(90.9-96.0)	0.3	(0.0-0.8)
Improving sleep habits	95.7	(93.3-97.6)	0.8	(0.0-1.9)
Participating in enjoyable activities	97.3	(95.7-98.9)	-	-
Getting out more	84.8	(81.0-88.2)	2.1	(0.8-3.7)

Help-seeking intentions and depression treatment beliefs amongst Sri Lankan Australians: A survey following a mental health literacy framework

Amanda Daluwatta, Kathryn Fletcher, Chris Ludlow & Greg Murray

Table S4 (Continued). Helpful and unhelpful ratings of each type of intervention (*N* = 374)

Interventions	Rated as "Helpful"		Rated as "Unhelpful"	
	%	(95% CIs)	%	(95% CIs)
Getting advice from family and friends	71.7	(67.4-76.5)	6.1	(3.7-8.6)
Getting hypnosis	8.6	(5.9-11.5)	42.5	(37.4-47.6)
Getting counselling or psychological therapy	97.1	(95.2-98.7)	0.8	(0.0-1.9)
Getting cognitive behavioural therapy	89.3	(85.8-92.5)	2.1	(0.8-3.7)
Talking to others who have faced similar problems	92.2	(89.6-94.9)	1.6	(0.5-2.9)
Improving eating habits	91.4	(88.5-94.1)	1.1	(0.3-2.1)
Undergoing electroconvulsive therapy	9.6	(6.7-12.6)	32.1	(27.3-37.2)
Cutting down on alcohol and cigarettes	86.6	(82.9-90.1)	5.3	(3.2-7.8)
Finding a support group for people with the same problem	89.6	(86.6-92.5)	2.9	(1.3-4.8)
Stopping going to work and staying at home	6.1	(3.7-8.6)	68.7	(63.9-73.3)
Using alcohol, cigarettes and drugs	2.7	(1.1-4.3)	90.9	(88.0-93.6)

Note: Percentages do not total to 100% due to not including the “don’t know” and “neither helpful nor unhelpful” responses
